# A Case Report of Comprehensive Diagnostic Approach on a Complex Left Adnexal Mass in a Young Female: Endometrioma or Complicated Ovarian Serous Cystadenofibroma

**DOI:** 10.7759/cureus.61798

**Published:** 2024-06-06

**Authors:** Rehab A Diab, Asmaa Eltobgy, Hala Adelhamied, Yasmeen Alabdallat

**Affiliations:** 1 Department of Medicine, Faculty of Medicine, Al-Azhar University, Cairo, EGY; 2 Department of Obstetrics and Gynecology, Faculty of Medicine for Girls, Al-Azhar University, Cairo, EGY; 3 Department of Clinical Pathology, Faculty of Medicine, Al-Azhar University, Cairo, EGY; 4 Faculty of Medicine, Hashemite University, Zarqa, JOR

**Keywords:** ovarian tumors, case report, endometrioma, serous cystadenofibroma, benign ovarian cyst

## Abstract

This case report presents a young female who was clinically, radiologically, and intraoperatively misdiagnosed as an ovarian endometrioma and was only diagnosed by histopathological biopsy as complicated serous cystadenofibroma, a rare benign tumor composed of both glandular and fibrous tissue. The diagnosis of adenofibroma typically involves a combination of imaging studies, such as ultrasound or magnetic resonance imaging (MRI) scan, and a histopathological biopsy to confirm the presence of the tumor. This case underscores the significance of utilizing various diagnostic methods and histopathological biopsies to diagnose and treat complex adnexal masses in females accurately.

## Introduction

Ovarian cysts are fluid-filled sacs that can form either within or on the surface of the ovary. These cysts may be benign or malignant. One type of benign ovarian cyst is the adenofibroma, constituting approximately 1.7% of the overall count of benign ovarian cysts [1]. They are rare cysts composed of both glandular and fibrous tissue, with the fibrous tissue being the predominant component [2,3]. Adenofibromas affect women of all age groups, with a higher incidence in women between the ages of 20 and 30 [4]. As the cyst grows larger, it can cause symptoms such as pelvic pain, pressure, vaginal bleeding, and dysuria [2]. Previous studies have reported sizes of up to 30 cm [5]. The risk of ovarian torsion, a gynecologic emergency, increases. Ultrasonography is pivotal in assessing serous cystadenofibroma and addressing concerns related to torsion [6]. Magnetic resonance imaging (MRI) has been documented as a valuable tool for distinguishing malignant tumors from benign ovarian lesions [7]. Cystadenofibromas can be difficult to diagnose due to their varied appearance in imaging studies, making it necessary to use a combination of imaging and tissue analysis to confirm the diagnosis. However, exploratory laparoscopy with the possibility of salpingo-oophorectomy is the most frequently employed treatment for serous cystadenofibroma [6]. This highlights the importance of utilizing multiple diagnostic modalities to accurately diagnose and treat complex adnexal masses in females and also highlights the difficulty in the differential diagnosis of complicated ovarian cysts and the value of histopathological diagnosis in such cases. Clinicians need to be aware of the complexities and nuances of ovarian cyst diagnosis and treatment to provide optimal care for affected patients.

## Case presentation

**Figure 1:**
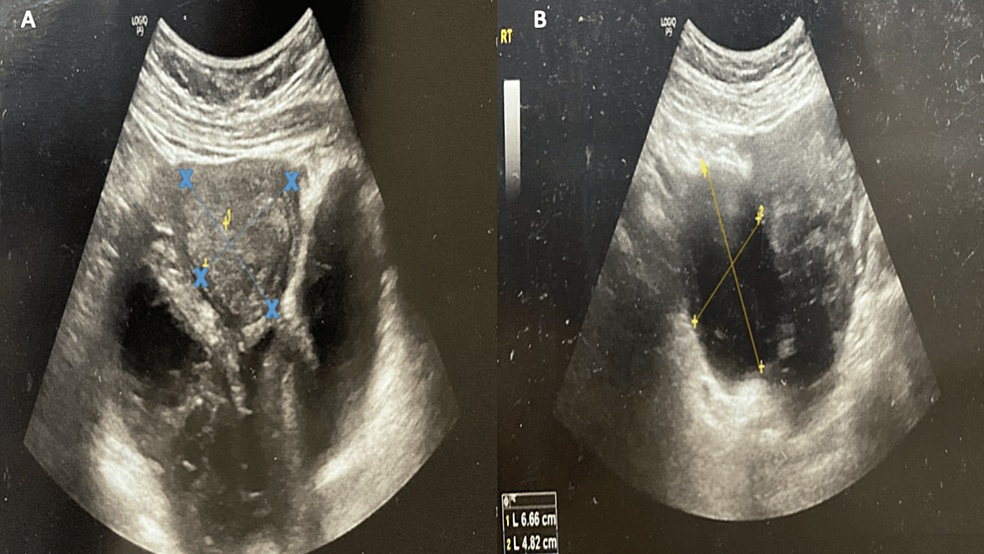
A transabdominal sonographic that shows a lesion on the left side (A), which is a complex, cystic, and multiloculated with several septations and echogenic material that appears to be retracting. It measures 5.92 x 4.74 cm and exhibits low vascularity. On the right side (B), a simple cyst is present with a thin wall. This cyst is characterized by hemorrhagic echogenicity and measures 6.6 x 4.8 cm. A minimal amount of free fluid is also detected in the pelvic area.

**Figure 2:**
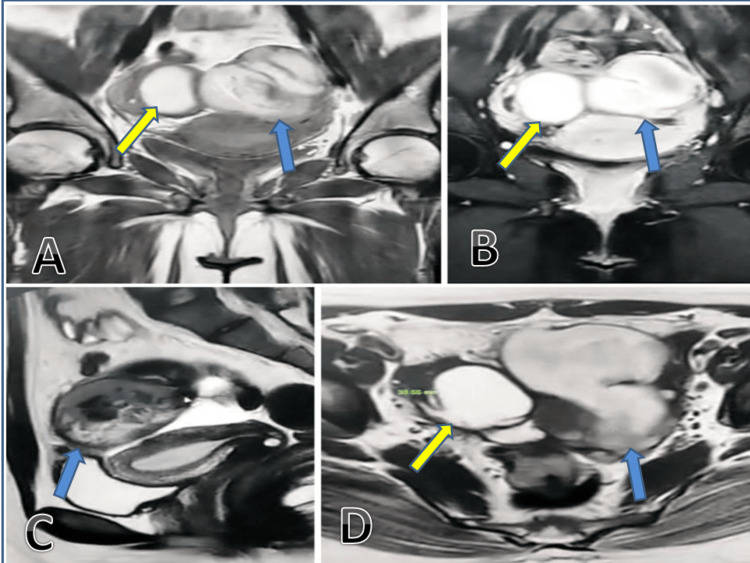
(A) Coronal T1W1, (B) coronal SPIR, (C) sagittal T2W1, and (D) axial T2W1. T1W1: T1-weighted image; SPIR: spectral presaturation with inversion recovery; T2W1: T2-weighted image The left side (blue arrows) shows a left adnexal, large, multiloculated complex heterogeneous lesion (98 mm x 62 mm x 59 mm) with cystic areas (low T1, high T2) and solid areas (heterogeneous intermediate T1 and T2) that enhance post-contrast. The right side (yellow arrows) shows a smaller cystic lesion (46 mm x 35 mm) in the right adnexal region with homogenous high T1, SPIR, and low T2 signals, plus a subcentimetric ovarian mural lesion with low signals in all sequences and no post-contrast enhancement.

**Figure 3:**
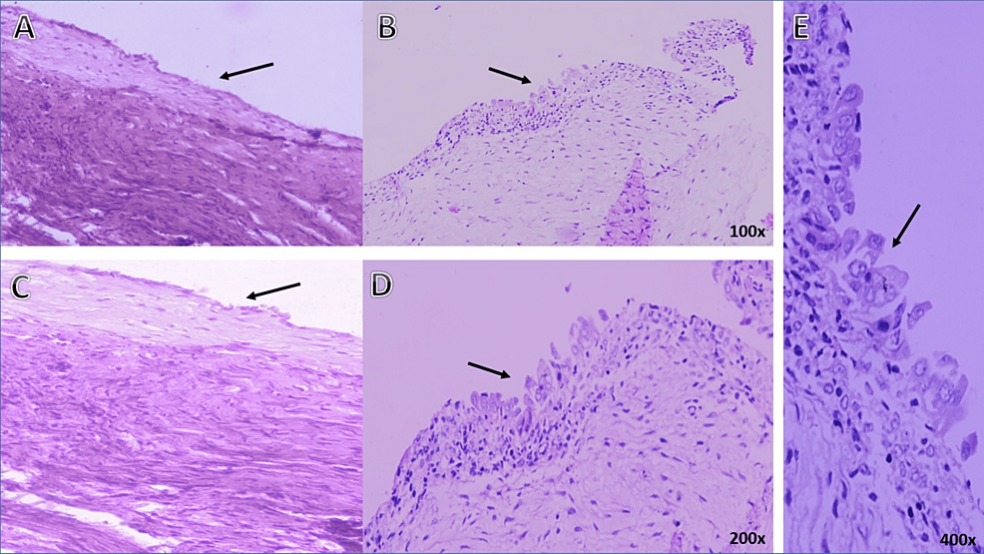
A histopathological picture of the left ovarian cyst wall obtained by ovarian cystectomy stained by hematoxylin and eosin (H&E), which is identified as a benign ovarian tumor. The tumor is lined by a type of epithelium known as ciliated pseudostratified cuboidal or columnar epithelium, as indicated by the arrows in images A, B, and C. This type of tumor is called a serous cystadenofibroma and is characterized by a prominent fibroblastic stromal component. The stroma is made up of spindle-shaped cells that are organized in fascicles and storiform patterns and is set in a background of collagen. Image B shows the tumor under 100x magnification power, image D shows it under 200x magnification power, and image E shows it under 400x magnification power. These images highlight the presence of Hobnail cells and variations in the amount of stromal cells present in the epithelium. The wall is partially devitalized and focally congested with extravasated red cells and hemosiderin. No evidence of malignancy was detected.

A 25-year-old virgin female presented to our gynecological unit with sudden-onset discomfort in the left lower quadrant, which had started one day prior. She had her last menstrual period three days ago, and there was no history of menstrual irregularities or previous surgical intervention. Initial laboratory tests, including a serum pregnancy test and urine analysis, were negative. Physical examination showed a lax abdomen with mild tenderness in the left iliac region. Pelvic ultrasound (Figure 1) revealed a complex cystic and solid left adnexal mass measuring 5.92 x 4.74 cm, with no separate identification of the left ovary, implying an ovarian origin. The mass showed minimal intralesional vascularity and no acoustic shadowing. The right side showed a simple hemorrhagic cyst measuring 4.5 x 4.2 cm, raising concerns regarding endometriosis (chocolate cyst) for further follow-up. There was minimal free fluid in the pelvis. The differential diagnosis included ovarian neoplasm, dermoid cyst, endometrioma (chocolate) cyst, acute pelvic inflammatory disease (PID), or tubo-ovarian abscess. The absence of constitutional manifestation of infection reduced the likelihood of acute PID. Further evaluation with tumor markers and MRI was recommended. The pelvic MRI (Figure 2) revealed a lobulated, multiloculated, and complex cystic mass measuring about 9.8 cm x 6.2 cm x 5.9 cm, with no separate identification of the left ovary. The cystic portion exhibited hypointensity on T1W and hyperintensity on T2W images, while the solid portion revealed intermediate signal intensities. The signal drop at spectral presaturation with inversion recovery (SPIR) weighted light signal (WLS) indicated a fatty component with heterogeneous enhancement in the post-contrast series. These features were suspicious for endometrioma or ovarian cystic neoplasm. A small quantity of free fluid was present in the pelvic cavity with no adjacent pelvic lymphadenopathy. Preoperative laboratory tests and tumor markers were performed. The results of cancer antigen (CA) 125, CA 19-9, and alpha-fetoprotein (AFP) were 49.6 U/ml (reference range: up to 39 U/ml), 279.4 U/ml (reference range: up to 35 U/ml), and 0 ng/ml (reference range: up to 40 ng/ml), respectively, reducing the concern for malignancy. The patient underwent an exploratory laparoscopic left ovarian cystectomy. Intraoperatively, the left side showed a well-circumscribed, smooth, thin-walled, bloody cystic mass measuring about 6 x 7 cm, suggestive of endometrioma (chocolate cyst), with mild adhesion to the ovarian wall. A frozen section was not available. The right side also showed a small hemorrhagic cyst measuring 3 x 4 cm. Based on an intraoperative gross picture, the patient was diagnosed with endometriosis. The blood was suctioned and the cyst wall was sent to histopathology. The histopathological diagnosis confirmed a left twisted benign ovarian cystadenofibroma (Figure 3). The patient was discharged and informed of a follow-up appointment for further assessment.

## Discussion

Serous cystadenofibroma is a type of ovarian neoplasm that belongs to the surface epithelial stromal tumor (SEST) category, which constitutes around two-thirds of overall ovarian tumors [8]. This tumor can impact females across different age groups, with a higher incidence among premenopausal women. A study by Jung et al. found that cystic adenofibroma is more common in premenopausal women [9]. Another study reviewed the clinicopathological features of cyst adenofibroma and found that the majority of cases are diagnosed in women under the age of 40 [10], which is consistent with our case, which was diagnosed at age 25 years old. The most commonly reported symptoms linked to serous cystadenofibroma include pelvic pain and discomfort, although other nonspecific symptoms such as vaginal bleeding, increased girth, and asymptomatic presentation can also occur [1]; however, there is a concern for additional complications such as ovarian torsion and dysfunctional uterine bleeding [11]. Dysfunctional uterine bleeding has been observed in our patient since the last attack. Ovulation disorder could also develop due to the hardened ovarian surface by solid tumor components and/or ovarian torsion. Imaging plays a crucial role in the diagnosis of serous cystadenofibroma. The standard imaging characteristics of this tumor might resemble those of a malignant neoplasm. However, the distinctive MRI appearance, often attributed to the fibrous component, provides a specific feature that aids in distinguishing it from malignant ovarian tumors [12]. On ultrasound (USG), a cystadenofibroma can exhibit a single or multiloculated cystic mass, accompanied by solid nodules or papillary projections [13]. However, USG cannot definitively characterize this tumor as its heterogeneous appearance mimics a malignant ovarian neoplasm or ovarian endometrioma. The assessment of ovarian tumors using MRI has been identified as a valuable method for distinguishing between malignant tumors and benign ovarian lesions [14]. Outwater et al. initially documented the MRI characteristic of cystadenofibroma, noting the low-signal intensity of the solid fibrous component in T2-weighted images [12]. This finding has been further elaborated in several other reports [15,16]. However, in our case, it was observed that the MRI exhibited high signal intensity on T2W for cystic components and intermediate intensity for solid components, which may be attributed to the bloody nature of the twisted cyst. The distinct presentation of blood products is indicated by relatively elevated signal intensity on T1W and intermediate to high signal intensity on T2W images. This similarity may pose challenges in differentiation from endometrioma, especially in the absence of prior imaging [17]. Serous cystadenofibroma typically occurs on one side, although there are instances where it may be observed in both ovaries [13]. A study by Yang et al. found that cystadenofibroma is more common in the ovary on the left side [10] which is in line with our case. Differentiation between benign and malignant tumors is often difficult on preoperative imaging, and a few cases have been reported as misdiagnosed as malignant tumors [1]. In our case, it was more difficult to diagnose on gross examination at the time of surgery, as the intraoperative macroscopic findings were similar to ovarian endometrioma. However, the final diagnosis of cystadenofibroma or endometrioma can only be confirmed by histological examination after surgery. In such cases, a frozen section diagnosis can be beneficial, potentially saving the patient from unnecessary extensive surgery by providing an accurate identification of cystadenofibroma in the operating room [15].

A frozen section was not done, which was a limitation in our case at the time of surgery. The histological identification of cystadenofibroma relied on the observation of a Müllerian epithelial lining in the cyst wall, accompanied by a significant presence of fibrous tissue in the underlying stroma [18]. Serous cystadenofibroma is predominantly cystic, with a clear, watery fluid or occasional blood filling and can present as an unilocular or multilocular mass [13]. Laterality is an important factor in determining the type of ovarian tumor, as bilateral presentation is more commonly associated with malignancy [8]. Serous cystadenofibroma is typically approached with two main treatment strategies. Given its benign nature, conservative management is recommended, and serial follow-up sonograms are advised [19]. Nonetheless, in cases where there is a concern for additional complications, the preferred and gold-standard treatment involves laparoscopic surgery for the complete removal of the tumor. This is particularly indicated when sonography confirms suspicions of malignancy or when there is a potential risk of rupture or ovarian torsion [6]. A case series by Czernobilsky et al. highlights the importance of histological examination for accurate diagnosis [18].

## Conclusions

In conclusion, adenofibromas represent a rare subtype of benign ovarian cysts that can affect women of all age groups. USG and MRI are useful tools in the evaluation of adenofibromas. However, due to the varied appearance of these cysts in imaging studies, a combination of imaging and tissue analysis is often used to confirm the diagnosis. The most common treatment for cystadenofibroma is exploratory laparoscopy with potential salpingo-oophorectomy. Considering the features of USG associated with the malignancy of this tumor and the potential for it to resemble an endometrioma during surgery, particularly in cases of torsion or congestion, It is prudent to consider the potential of a benign tumor before opting for an aggressive surgical approach or hormonal therapy. This case underscores the significance of employing various diagnostic methods to diagnose and treat complex adnexal masses in females accurately and the difficulty in differentiating complicated ovarian cysts. Additionally, the importance of using multiple diagnostic methods and considering a patient's symptoms and medical history is emphasized in this case involving complex adnexal masses in females, particularly in distinguishing complicated ovarian cysts. It is crucial to use multiple imaging techniques for proper identification and diagnosis to ensure the best treatment plan and minimize potential complications.

## References

[1] Cho DH (2018). Serous cystadenofibroma misdiagnosed as an ovarian malignancy. BMJ Case Rep.

[2] Compton HL, Finck FM (1970). Serous adenofibroma and cystadenofibroma of the ovary. Obstet Gynecol.

[3] McNulty JR (1959). The ovarian serous cystadenofibroma; a report of 25 cases. Am J Obstet Gynecol.

[4] Desmond M, Baun J (2015). Ovarian torsion with persistent parenchymal blood flow. J Diagn Med Sonogr.

[5] Virgilio BA, De Blasis I, Sladkevicius P (2019). Imaging in gynecological disease (16): clinical and ultrasound characteristics of serous cystadenofibromas in adnexa. Ultrasound Obstet Gynecol.

[6] Krohn KA (2021). Sonographic evaluation of serous cystadenofibroma with evidence of intermittent torsion. J Diagn Med Sonogr.

[7] Ma FH, Zhao SH, Qiang JW, Zhang GF, Wang XZ, Wang L (2014). MRI appearances of mucinous borderline ovarian tumors: pathological correlation. J Magn Reson Imaging.

[8] Modepalli N, Venugopal SB (2016). Clinicopathological study of surface epithelial tumours of the ovary: an institutional study. J Clin Diagn Res.

[9] Jung DC, Kim SH, Kim SH (2006). MR imaging findings of ovarian cystadenofibroma and cystadenocarcinofibroma: clues for the differential diagnosis. Korean J Radiol.

[10] Yang S, Yang Z, Zhang S, Len T, Yang L (2020). Coexistence of genital tuberculosis and ovarian serous cystadenofibroma in a young female patient: a case report. J Int Med Res.

[11] Hibbard LT (1985). Adnexal torsion. Am J Obstet Gynecol.

[12] Outwater EK, Siegelman ES, Talerman A, Dunton C (1997). Ovarian fibromas and cystadenofibromas: MRI features of the fibrous component. J Magn Reson Imaging.

[13] Alcázar JL, Errasti T, Mínguez JA, Galán MJ, García-Manero M, Ceamanos C (2001). Sonographic features of ovarian cystadenofibromas: spectrum of findings. J Ultrasound Med.

[14] Zhang H, Zhang GF, Wang TP, Zhang H (2013). Value of 3.0 T diffusion-weighted imaging in discriminating thecoma and fibrothecoma from other adnexal solid masses. J Ovarian Res.

[15] Cho SM, Byun JY, Rha SE (2004). CT and MRI findings of cystadenofibromas of the ovary. Eur Radiol.

[16] Jung SE, Lee JM, Rha SE, Byun JY, Jung JI, Hahn ST (2002). CT and MR imaging of ovarian tumors with emphasis on differential diagnosis. Radiographics.

[17] Nishi M, Akamatsu N, Sekiba K (1990). Magnetic resonance imaging of the ovarian cyst: its diagnostic value of endometrial cyst. Med Prog Technol.

[18] Czernobilsky Czernobilsky, B. B., Borenstein Borenstein, R. & Lancet, M M (1974). Cystadenofibroma of the ovary: a clinicopathologic study of 34 cases and comparison with serous cystadenoma. Cancer.

[19] Adilgereyeva AS, Abdelazim IA, Zhurabekova GA (2019). Clinical and pathological features of women with adnexal masses admitted as emergency cases to the Gynaecology Department of West Kazakhstan University. Prz Menopauzalny.

